# Case Report: Cardiac metastasis from urothelial carcinoma with predominant squamous differentiation in a young woman: first reported case treated with enfortumab vedotin plus pembrolizumab

**DOI:** 10.3389/fonc.2026.1786375

**Published:** 2026-05-20

**Authors:** Alix Vansteeger, El Mamoune El Ghali Benlemlih, Antoine Delmarche, Stéphane Thiry, Hélène Dano, Justine Castaigne, Vassiliki Pasoglou, Sandy Van Nieuwenhove, Bernhard Gerber, Gaby Aphram, Qaid Ahmed Shagera, Christophe Beauloye, Rachel Galot, Cédric van Marcke, Emmanuel Seront

**Affiliations:** 1 Institut Roi Albert II, Medical Oncology, Cliniques universitaires Saint-Luc, Brussels, Belgium; 2 Department of Urology, Cliniques universitaires Saint-Luc, Brussels, Belgium; 3 Department of Pathology, Cliniques universitaires Saint-Luc, Brussels, Belgium; 4 Department of Radiology, Cliniques universitaires Saint-Luc, Brussels, Belgium; 5 Cardiovascular Department, Cliniques universitaires Saint-Luc, Brussels, Belgium; 6 Department of Cardiovascular Surgery, Cliniques universitaires Saint-Luc, Brussels, Belgium; 7 Department of Nuclear Medicine, Cliniques universitaires Saint-Luc, Brussels, Belgium

**Keywords:** cardiac metastasis, enfortumab-pembrolizumab, squamous differentiation, surgery, urothelial carcinoma

## Abstract

Cardiac metastases from urothelial carcinoma (UC) are rare and have a poor prognosis. A 36-year-old woman presented with *de novo* metastatic muscle-invasive bladder cancer, bulky retroperitoneal lymphadenopathy, and a large right ventricular mass nearly obliterating the cavity and extending to the pulmonary artery. Because of impending right ventricular outflow tract obstruction, urgent surgical debulking was performed; a friable tumor encasing the tricuspid valve filled the right ventricle, valve preservation was not feasible, and postoperative pacing was required. Transurethral resection of the bladder tumor showed high-grade UC with predominant (80%) squamous differentiation, while the cardiac lesion demonstrated a pure squamous phenotype with immunohistochemistry supporting urothelial origin. Given a very high tumor burden and postoperative cisplatin ineligibility, first-line enfortumab vedotin plus pembrolizumab was initiated, but the patient rapidly deteriorated with early radiologic progression and died ~8 weeks after diagnosis. This case illustrates the aggressive course of squamous-dominant metastatic UC with intracardiac involvement, the therapeutic trade-off of urgent cardiac surgery, and the limited evidence for enfortumab vedotin-based regimens in squamous-predominant disease.

## Highlights

- Cardiac UC metastasis, although rare, may be seen on staging CT—systematically review the heart and great vessels, even with mild symptoms.
- If suspected, obtain rapid echo ± cardiac CT/MRI to assess hemodynamic risk and differentiate tumor vs thrombus; ^18^F-FDG PET/CT can help when equivocal.
- With impending RV outflow obstruction, urgent debulking can be life-saving and diagnostic, but may worsen performance status and limit systemic options.
- Squamous shift (squamous-dominant primary, pure squamous metastasis) suggests clonal selection and very aggressive biology.
- Enfortumab vedotin–pembrolizumab is first-line in metastatic UC, but activity in pure/predominantly squamous disease and cardiac metastasis remains unclear.

## Introduction

Cardiac metastases from solid tumors are rare, occurring in approximately 6%–20% of autopsies in patients with carcinoma (1). Only a small proportion arises from urothelial carcinoma (UC), and fewer than 100 cases have been reported over nearly a century (1). Reported patients are typically men older than 50 years, most often with recurrent or previously treated bladder cancer, and outcomes are dismal, with survival after diagnosis ranging from days to roughly 1 year (2).

Cardiac involvement poses specific and understudied challenges. First, cardiac metastases may present with non-specific respiratory or constitutional symptoms, leading to diagnostic delay despite the potential for rapid hemodynamic deterioration (2). Second, management often requires a difficult trade-off between urgent local intervention and the need to rapidly initiate systemic therapy. In particular, major cardiac surgery in a palliative metastatic setting, while sometimes unavoidable, may entail substantial morbidity, delay systemic treatment, and impair performance status—potentially compromising eligibility for cisplatin-based chemotherapy and narrowing therapeutic options.

Here, we report a young woman with *de novo* metastatic muscle-invasive bladder cancer and a large right ventricular mass. The primary tumor showed UC with predominant squamous differentiation, whereas the resected cardiac metastasis demonstrated a pure squamous phenotype with immunohistochemistry supporting urothelial origin. We discuss the implications for tumor biology, emphasize the need for cardiac imaging in patients with new or unexplained cardiopulmonary symptoms—particularly when squamous differentiation is prominent—and consider the strategic balance between cardiac debulking and systemic treatment sequencing.

## Case description

### Patient

A 36-year-old woman with hypertension, obesity, and a 20-pack-year smoking history underwent routine gynecologic ultrasonography, which incidentally revealed an intravesical polypoid lesion. She had no urinary or cardiorespiratory symptoms at that time. Her medical history was notable for a psychotic disorder treated with lithium for 20 years.

Transurethral resection of the bladder tumor (TURBT), performed at an outside institution, demonstrated high-grade muscle-invasive UC with predominant (80%) squamous differentiation. A staging contrast-enhanced computed tomography (CT) of the chest, abdomen, and pelvis performed at that institution reported bulky nodal disease involving the internal and external iliac, obturator, and retroperitoneal stations, while no cardiac abnormality was described. She was referred to our center and reported mild progressive exertional dyspnea (New York Heart Association (NYHA) class II). On review of the baseline CT, a large right ventricular intracardiac mass was identified in addition to bulky abdominopelvic lymphadenopathy (**Figures 1A, B**). Laboratory testing revealed a mild inflammatory syndrome and elevated N-terminal pro-B-type natriuretic peptide (NT-proBNP; 2,214 pg/mL) without troponin elevation; renal function was preserved (creatinine clearance >60 mL/min). Transthoracic echocardiography and cardiac magnetic resonance imaging (MRI) identified a large intracavitary mass occupying the entire right ventricle and outflow tract, infiltrating the free wall and interventricular septum, with extension to the pericardium—features favoring malignancy over thrombus (**Figures 1C, D**). Whole-body ^18^F-fluorodeoxyglucose positron emission tomography (PET)-CT demonstrated intense uptake in the cardiac mass and multiple abdominal and pelvic lymph nodes, consistent with disseminated metastatic disease (**Figure 2**).

**Figure 1:**
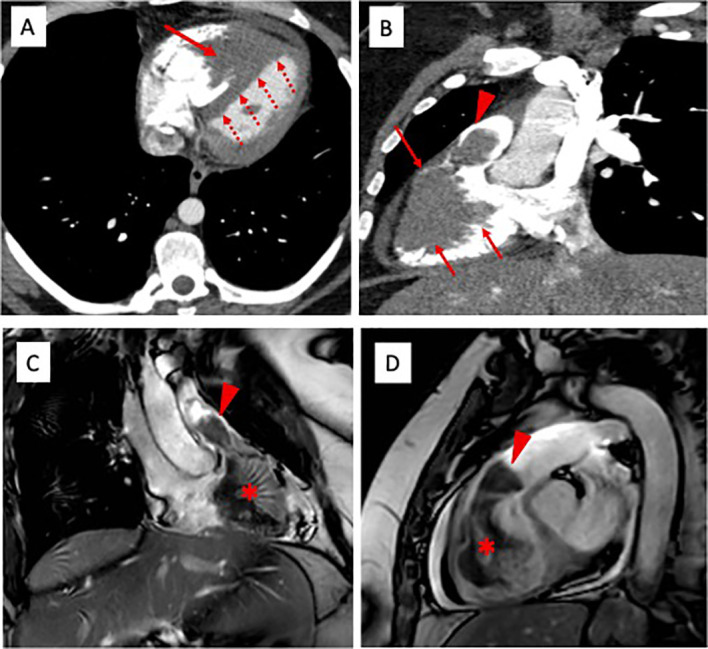
**(A, B)** Computed tomography: axial **(A)** and sagittal **(B)** images from a subsequent non-cardiac-gated, contrast-enhanced chest CT demonstrate a large hypoattenuating mass within the right ventricle (arrows), extending into the pulmonary artery (arrowheads) and infiltrating the ventricular wall (dotted arrows). **(C, D)** Cardiac MRI cine balanced single-shot balanced turbo field echo (SBTFE) in coronal **(C)** and sagittal **(D)** confirms the right ventricular mass (*) and its mobile component extending toward the pulmonary artery (arrowhead). Tissue characterization (T2, first-pass perfusion, and late gadolinium enhancement) is reported as heterogeneous with a centrally hypoperfused core.

**Figure 2:**
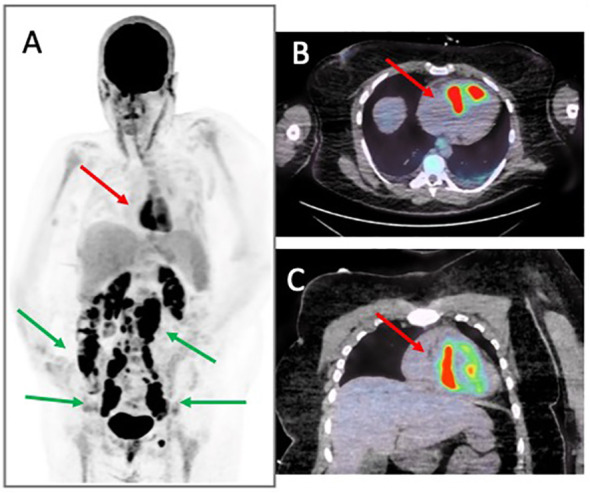
Maximum intensity projection (MIP), coronal **(A, C)**, and axial **(B)** ^18^F-FDG PET/CT images showing a right intraventricular mass with intense, heterogeneous FDG uptake (red arrows), along with multiple abdominal and pelvic lymph nodes demonstrating intense hypermetabolic activity (green arrows).

### Timeline

Given the imminent risk of hemodynamic collapse due to right ventricular outflow tract obstruction, urgent surgical debulking was performed. Intraoperatively, a large friable tumor encased the tricuspid valve and filled the right ventricle. The lesion was tightly adherent to the ventricular wall, precluding “en bloc” resection, and tricuspid valve preservation was not feasible. A replacement with a bioprosthesis was performed. Tumor extension toward the pulmonary valve and into the pulmonary artery was also noted. The postoperative course was complicated by complete atrioventricular block requiring permanent pacemaker implantation.

### Diagnosis

Histological examination of the cardiac specimen showed infiltration by a moderately differentiated, keratinizing squamous cell carcinoma (100%) with extensive necrosis. Immunohistochemistry demonstrated nuclear expression of p40 and GATA3 in the tumor cells, while p16 was negative; CK5 staining was non-contributory (**Figure 3**). Integrating these findings with the bladder specimen, and in the absence of another suspected primary tumor, the final diagnosis was high-grade muscle-invasive bladder UC with predominant squamous differentiation, infra-diaphragmatic nodal metastases (multiple abdominal and pelvic lymph nodes), and a cardiac metastasis displaying purely squamous histology.

**Figure 3:**
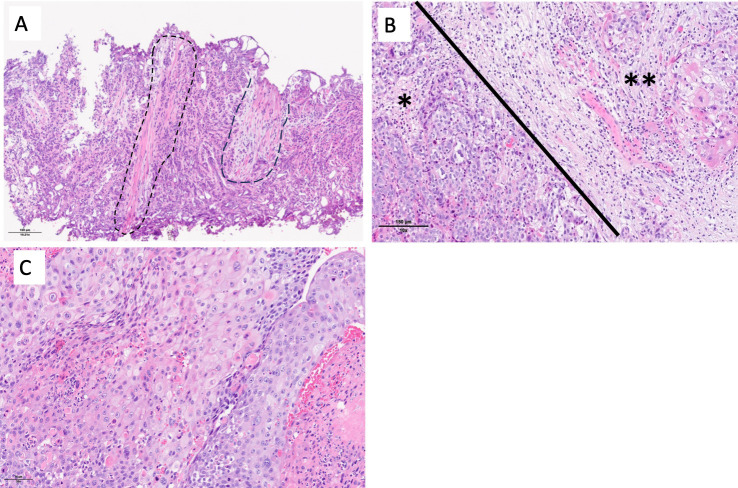
**(A)** Tissue sample from bladder: urothelial carcinoma with smooth muscle fiber infiltration (encircled). **(B)** Urothelial tumor with predominant squamous component (>80%). The minor urothelial is located in the left part (*), while the squamous component is on the right (**). **(C)** Tissue sample from cardiac surgery: pure epidermoid tumor component consisting of large tumor nests, sometimes fused into cohesive areas.

### Treatment

The final stage was cT2 cN3 pM1b, stage IVB. Radical cystectomy was not performed because of extensive metastatic disease at diagnosis, and systemic treatment was therefore prioritized. After cardiac surgery, the patient was ineligible for cisplatin-based chemotherapy because of a marked decline in performance status (creatinine clearance 40 mL/min), together with postoperative renal impairment. As the obstructive intracardiac lesion had been removed and the immediate hemodynamic risk controlled, enfortumab vedotin (EV) plus pembrolizumab (P) was initiated as first-line systemic therapy; platinum-based chemotherapy was reserved as a potential subsequent option should clinical and renal status improve. EV (1.25 mg/kg) and pembrolizumab (200 mg) were started 5 days postoperatively, approximately 4 weeks after TURBT.

### Outcome

Within days, her condition deteriorated rapidly. Although the EV dose at day 8 was administered, her performance status continued to decline. She developed a fluctuating confusional state with apathy, agitation, and disorientation, associated with severe diffuse pain, profound asthenia, and inferior limb edema. Neurological examination revealed no focal deficits and no objective loss of strength or sensation. Serum lithium was slightly above the therapeutic range, prompting dose adjustment. EEG showed no epileptiform activity. Brain MRI was contraindicated because of the recently implanted pacemaker; contrast-enhanced brain CT was unremarkable. Cerebrospinal fluid analysis showed only a few white blood cells, with no microbiological evidence of infection and no malignant cells. Repeat echocardiography showed no evidence of endocarditis.

Restaging CT performed 18 days after treatment initiation demonstrated diffuse radiologic progression, but no early recurrence of the cardiac mass. Broad-spectrum antibiotics and corticosteroids were initiated for suspected neurological immune-related adverse events despite the very early onset after P exposure; however, given the timing and concomitant tumor progression, causality could not be established. Overall, the confusional syndrome was considered multifactorial (including rapid malignant progression and possible contributory metabolic/drug-related factors in a patient with a pre-existing psychiatric disorder treated with lithium). Ongoing clinical deterioration precluded further systemic therapy, including continuation of the second cycle of EV–P or a new line with platinum-based chemotherapy. She died 21 days after starting EV–P, approximately 2 months after the initial diagnosis. Autopsy was declined by the family.

## Discussion

This case highlights the fulminant course of squamous-dominant metastatic UC, presenting with bulky nodal disease and a massive right ventricular metastasis, followed by rapid systemic progression despite early EV–P. Although the intracardiac lesion was locally controlled, the overall trajectory suggests that cardiac involvement may be less a direct driver of outcome once treated and more a marker of highly aggressive systemic disease.

The diagnostic aspect deserves emphasis. In our patient, the right ventricular mass was not mentioned in the initial report (although visible), underscoring the importance of systematic assessment of the cardiac chambers and great vessels on routine staging CT, even when cardiopulmonary symptoms are mild. Intracardiac metastases may mimic non-specific respiratory complaints yet rapidly become fatal (2). When a cardiac lesion is suspected or identified on CT, prompt dedicated evaluation (echocardiography and CT/MRI when feasible) is warranted to define hemodynamic risk and guide urgent management (3–5). In addition, ^18^F-FDG PET-CT may support a malignant etiology and help distinguish tumor from bland thrombus when conventional imaging is equivocal (2, 6–9).

Therapeutically, urgent cardiac surgery in this setting is mainly life-saving/palliative and diagnostic, intended to relieve obstruction and obtain tissue rather than to alter the systemic trajectory (2–5, 10–12). In our patient, debulking controlled the immediate hemodynamic threat; however, major surgery in a palliative metastatic context may be followed by postoperative complications and functional decline, which can delay systemic therapy, limit treatment continuation, and reduce overall therapeutic flexibility (11). In our case, a severe confusional syndrome occurred early in the postoperative period; a multifactorial etiology was considered (including major surgery, concomitant medications, metabolic factors, rapidly progressive malignancy, and the patient’s pre-existing psychiatric disorder), and a direct contribution of surgery to neuropsychiatric vulnerability cannot be excluded. Long-term lithium exposure may also have contributed to reduced renal reserve and increased vulnerability to metabolic or neurocognitive complications, particularly in the postoperative setting. However, there is no evidence to suggest that the patient’s psychiatric history or lithium treatment directly influenced tumor biology or disease aggressiveness. Whether an initial non-surgical strategy with immediate systemic therapy would have altered the outcome remains speculative.

Pathology across sites was informative and supports a continuum toward complete squamous differentiation in the metastatic compartment. The bladder tumor showed UC with predominant (>80%) squamous differentiation, whereas the resected intracardiac lesion displayed a pure squamous phenotype. This pattern is compatible with metastatic seeding by a squamous-dominant clone within a heterogeneous primary and/or progressive clonal selection during dissemination. Importantly, squamous differentiation in UC is generally associated with more aggressive clinicopathological features, including advanced-stage disease, higher tumor burden, and poorer prognosis, which may partly explain the fulminant clinical course observed in our patient (13). Although tissue confirmation of cardiac lesions is uncommon and selection bias is likely (**Table 1**), published UC cardiac metastases with documented histology frequently report squamous features (2–4, 6, 10, 12), raising the hypothesis that squamous-dominant disease may be associated with cardiac involvement—either through a specific tropism or as a surrogate of extreme aggressiveness (9, 14–17). Across reports, outcomes remain uniformly poor, with survival often limited to hours to a few months (2).

**Table:** TABLE 1 Outcomes of patients with cardiac metastasis from urothelial carcinoma.

Ref	No. of cases	Age (years) Sex m/f	Metachronous vs synchronous cardiac metastasis (interval duration)	Cardiac tissue biopsy	Histology of bladder (B) and cardiac (C) tissue	Treatment for cardiac metastasis Best response	OS (months)
(2)	30	Age = 42–77 Sex = m (14)/f (4)/na (12)	Metachronous (n = 13, from 4 to 204 months) Autopsy (n = 15) NA (n = 2)	4 biopsies and 1 surgery	B = 29 with UC and 1 UC with squamous diff C = 5 samples, all UC	Surgical resection (1) Radiotherapy (2) Chemotherapy (6) Immunotherapy (2)	5 hours to 360 days
(1)	1	66/m	Metachronous (7 yr)	None	B = UC	None	0.5
(11)	1	75/m	Metachronous (7 yr)	Surgery	B = Poorly differentiated UC C = Poorly differentiated UC	Surgical resection	0.5
(14)	1	64/m	Metachronous (3 mo)	None	B = UC	Tislelizumab (PR)	NA
(15)	1	58/m	Metachronous (6 mo)	None	B = UC	Pembrolizumab (CR)	NA
(19)	1	74/f	Metachronous (39 mo)	None	B = UC	Enfortumab vedotin (CR)	NA
(9)	1	67/m	Metachronous (12 mo)	None	B = UC	Cisplatin–gemcitabine followed by avelumab (PD)	2
(16)	1	62/m	Metachronous (9 mo)	None	B = UC with squamous diff	Carboplatin–gemcitabine (PD)	<12 months
(17)	1	51/f	Synchronous	None	B = UC with squamous cell diff	Cisplatin–gemcitabine (SD)	<12 months
(4)	1	69	Metachronous (NA)	Surgery	B = UC C = UC with squamous diff	Surgical resection followed by cisplatin-based chemotherapy (NA)	3
(12)	1	58/f	Synchronous	Surgery	B = UC C = UC with squamous diff	Surgical resection followed by cisplatin–gemcitabine (NA)	No recurrence at 9 weeks
(6)	1	51/m	Metachronous (12 mo)	Biopsy	B = UC C = UC with squamous diff	Radiotherapy and atezolizumab (SD)	8.5
(3)	1	64/m	Metachronous (7 mo)	Biopsy and surgery	B = UC C = UC with squamous diff	Surgical resection Platinum-based chemotherapy (PD)	1.5
(7)	1	73/m	Metachronous (6 yr)	None	B = UC with squamous diff	Cisplatin–gemcitabine (SD)	8
(8)	1	55/f	Metachronous (6 mo)	None	B = Squamous cell carcinoma	NA	NA
(10)	1	79/m	Metachronous (4 mo)	Biopsy	B = UC with squamous diff C = Squamous cell carcinoma	Radiotherapy (PD)	NA

Ref= reference; m, male; f, female; NA, not available; yr, years; mo, months; B, bladder; C, cardiac; UC, urothelial carcinoma; diff, differentiation; CR, complete response; PR, partial response; SD, stable disease; OS, overall survival.

EV–P has become a first-line standard in metastatic UC, but its activity in pure squamous carcinoma remains less well defined. In the UNITE retrospective cohort, objective response rates were broadly similar for squamous-dominant UC (50%–99% squamous component) and conventional UC (<50% squamous component) (56% and 62%, respectively), whereas responses were markedly lower in pure squamous carcinoma (17%) (18). In our case, postoperative frailty and the urgency to initiate systemic treatment supported EV–P as the most reasonable option; nevertheless, the patient experienced very early radiologic progression and died shortly after treatment initiation. Beyond the possibility of intrinsically reduced sensitivity in a squamous-predominant metastatic clone, several factors could plausibly explain the apparent lack of benefit, including extreme tumor burden, explosive growth kinetics, and very short effective exposure to therapy due to rapid clinical deterioration.

Overall, despite advances in first-line therapy for metastatic UC, the effectiveness of EV–P in the rare setting of cardiac metastasis—particularly in squamous-dominant disease—remains insufficiently characterized (19). Systematic review of the heart on staging CT may be particularly important in squamous-dominant UC, where aggressive biology may compress the window for life-saving hemodynamic stabilization.

## Conclusion

In conclusion, squamous-dominant metastatic UC can present with massive intracardiac involvement and an extremely aggressive clinical course. Careful review of the heart on routine staging CT—followed by prompt echocardiography and cardiac CT/MRI when a lesion is suspected—is essential to identify patients at risk of rapid hemodynamic collapse. When urgent cardiac debulking is required, postoperative morbidity may narrow systemic options, and the benefit of EV–pembrolizumab in squamous-predominant disease with cardiac metastasis remains insufficiently defined.

## Patient perspective

The family raised awareness of intracardiac metastases as a potential early manifestation of aggressive urothelial carcinoma and highlighted the diagnostic and therapeutic challenges posed by impending cardiac outflow obstruction in a metastatic setting.

## Data Availability

The original contributions presented in the study are included in the article/supplementary material. Further inquiries can be directed to the corresponding author.

## References

[1] Murakami T Komiya A Mikata K Kaneko S Ikeda I . Cardiac metastasis of renal pelvic cancer. Int J Urol. (2007) 14:240–1. doi: 10.1111/j.1442-2042.2007.01512.x. PMID: 17430263

[2] de Araujo Souza LC Ribeiro EC Pinto TDA de Ulhoa Barbosa TMJ da Trindade Netto MB Barbosa SAC . Urothelial bladder cancer with cardiac metastasis: Literature review and case report. Int J Surg Case Rep. (2023) 111(2):4–7. doi: 10.1016/j.ijscr.2023.108630. PMID: 37708786 PMC10507135

[3] Zanchettin C Zanella L Zoffoli G Venturini A Gallingani A Ronco F . Ultra-rapid regrowth of urothelial cardiac metastasis after debulking surgery. Cardiovasc Pathol. (2021) 53:2–4. doi: 10.1016/j.carpath.2021.107334. PMID: 33753239

[4] Paiva MS Rocha BM Albuquerque F Abecasis J . Restaging of a urothelial tumour from a rare cardiac metastasis: a multidisciplinary challenge. Eur Heart J Case Rep. (2025) 9:1–2. doi: 10.1093/ehjcr/ytaf465. PMID: 41132586 PMC12540022

[5] Mountzios G Bamias A Dalianis A Danias P Pantelidaki E Nanas J . Endocardial metastases as the only site of relapse in a patient with bladder carcinoma: A case report and review of the literature. Int J Cardiol. (2010) 140:e4. doi: 10.1016/j.ijcard.2008.11.012. PMID: 19046610

[6] Arscott WT Lal P Mamtani R O’Quinn R Deo R Jones J . Long-term survival after treating cardiac metastasis with radiation and immune therapy: A case report. Cureus. (2018) 10(5):e2607:2-3. doi: 10.7759/cureus.2607. PMID: 30013871 PMC6039219

[7] Nakashima Y Tanioka K Kubo T Yamasaki N Yamasaki I Syuin T . Metastatic cardiac tumor from urothelial carcinoma detected by transthoracic echocardiography: A case report. J Med Case Rep. (2015) 9:2–4. doi: 10.1186/s13256-015-0740-3. PMID: 26567955 PMC4645472

[8] Gorospe L García-Santana E Jane-Soler P Gómez-Martínez MV Gómez-Dos-Santos V García-Gómez-Muriel I . Cardiac and skeletal muscle metastases from squamous cell carcinoma of the bladder: Positron emission tomography-computed tomography findings. Urology. (2017) 100:e5–6. doi: 10.1016/j.urology.2016.11.030. PMID: 27890686

[9] Teixeira AR Vaz-Ferreira A Carneiro F Sousa JP . Cardiac metastasis of bladder carcinoma: case report and literature review. Rev Portuguesa Oncologia. (2025) 8:25–8. doi: 10.24875/rpo.m25000002

[10] White WM Waters WB Panella TJ Klein FA . Metastasis of squamous cell carcinoma of the bladder to the heart: Case report and review of the literature. Urology. (2006) 68:890.e9–890.e10. doi: 10.1016/j.urology.2006.05.004. PMID: 17070384

[11] Sobczyk D Nosal M Myc J Trybowski G Gorkiewicz-Kot I Olejniczak P . Cardiac metastasis due to pulmonary metastasis from a transitional cell carcinoma. Eur J Echocardiography. (2008) 9:113–5. doi: 10.1016/j.euje.2007.03.042. PMID: 17616442

[12] Shields AM Pomplun S Deshpande R Whitaker DC . Right ventricular metastasis of transitional cell carcinoma of the renal pelvis: Successful single stage surgical treatment. Interact Cardiovasc Thorac Surg. (2011) 12:297–300. doi: 10.1510/icvts.2010.256230. PMID: 21051378

[13] Laymon M Mosbah A Hashem A Mahmoud O Harraz AM Elsawy AA . Oncologic outcomes of squamous cell carcinoma versus urothelial carcinoma with squamous differentiation after radical cystectomy for bladder carcinoma. Clin Genitourin Cancer. (2022) 20:148–54. doi: 10.1016/j.clgc.2021.12.011. PMID: 34998698

[14] Zhu L Li Z Wang Z Chen J Zhang H Zhao X . A rare case of bladder cancer that metastasized to brain, heart, and lung lymph nodes benefited from immunotherapy. World J Surg Oncol. (2022) 20:2–7. doi: 10.1186/s12957-022-02876-9. PMID: 36529739 PMC9762084

[15] Chan HY Chan WY Lin JWS . Cardiac and intramuscular metastases following nephroureterectomy for metachronous urothelial carcinoma. Am J Case Rep. (2024) 25:2–5. doi: 10.12659/AJCR.942864. PMID: 38650318 PMC11056213

[16] Al-Bitar A Kouli A Talaba J Azarpayk M Al Tawil M . Cardiac metastasis in upper tract urothelial carcinoma with squamous differentiation. Case Rep Oncol. (2025) 18(1):763–72. doi: 10.1159/000546330. PMID: 40534730 PMC12176369

[17] Duwe G Fischer ND Haack M Höfner T Haferkamp A Brandt MP . Solitary cardiac metastasis of urothelial carcinoma of the urinary bladder with squamous cell differentiation – a rare manifestation. Urol Case Rep. (2023) 46. doi: 10.1016/j.eucr.2023.102318. PMID: 36632283 PMC9827352

[18] Koshkin VS Henderson N James M Natesan D Freeman D Nizam A . Efficacy of enfortumab vedotin in advanced urothelial cancer: Analysis from the Urothelial Cancer Network to Investigate Therapeutic Experiences (UNITE) study. Cancer. (2022) 128:1194–205. doi: 10.1002/cncr.34057. PMID: 34882781

[19] Tokitaka S Hirata H Shimo R Shintaro O Shiraishi K . Effectiveness of enfortumab-vedotin for right atrial metastasis following total cystectomy: A case report. Cureus. (2024) 16(12):e75122:1–3. doi: 10.7759/cureus.75122. PMID: 39759708 PMC11698909

